# Analytical technologies for influenza virus-like particle candidate vaccines: challenges and emerging approaches

**DOI:** 10.1186/1743-422X-10-141

**Published:** 2013-05-04

**Authors:** Christine M Thompson, Emma Petiot, Alexandre Lennaertz, Olivier Henry, Amine A Kamen

**Affiliations:** 1 National Research Council Canada, Vaccine Program – Human Health therapeutics Portfolio, 6100 Royalmount Avenue, Montreal, Québec H4P 2R2, Canada; 2 École Polytechnique de Montréal, C.P. 6079, succ. Centre-ville, Montréal, Québec H3C 3A7, Canada

**Keywords:** Influenza virus-like particles (VLPs), Quantification, Process Analytical Technologies, Total particles, Vaccines

## Abstract

Influenza virus-like particle vaccines are one of the most promising ways to respond to the threat of future influenza pandemics. VLPs are composed of viral antigens but lack nucleic acids making them non-infectious which limit the risk of recombination with wild-type strains. By taking advantage of the advancements in cell culture technologies, the process from strain identification to manufacturing has the potential to be completed rapidly and easily at large scales. After closely reviewing the current research done on influenza VLPs, it is evident that the development of quantification methods has been consistently overlooked. VLP quantification at all stages of the production process has been left to rely on current influenza quantification methods (i.e. Hemagglutination assay (HA), Single Radial Immunodiffusion assay (SRID), NA enzymatic activity assays, Western blot, Electron Microscopy). These are analytical methods developed decades ago for influenza virions and final bulk influenza vaccines. Although these methods are time-consuming and cumbersome they have been sufficient for the characterization of final purified material. Nevertheless, these analytical methods are impractical for in-line process monitoring because VLP concentration in crude samples generally falls out of the range of detection for these methods. This consequently impedes the development of robust influenza-VLP production and purification processes. Thus, development of functional process analytical techniques, applicable at every stage during production, that are compatible with different production platforms is in great need to assess, optimize and exploit the full potential of novel manufacturing platforms.

## Introduction

The World Health Organization (WHO) reports approximately 500 million cases of influenza infection and between 250,000 to 500,000 deaths annually due to seasonal epidemics [1,2]. To combat the persistent threat of influenza epidemics, vaccination remains the most efficient strategy to prevent infection. Since the 1950s, egg-based production processes remain the standard method to produce seasonal influenza inactivated whole, split, subunit or live-attenuated vaccines. The influenza H1N1 pandemic of 2009 has clearly highlighted the limitations of the current egg-based production methods in the case of the emergence of a pandemic strain [3,4]. The main drawback is the relatively long 6-month period from strain isolation to final dose formulation and validation [5]. Moreover, in 2006, WHO published an action plan to increase the current supply of influenza vaccine. One of the goals was to reach 2340 million monovalent doses produced in case of a global pandemic, which highlights the need to develop new technologies capable to support urgent and large demands for vaccines [6]. As a result, strategies to shorten the response time for vaccine preparation and to expand production capacity are increasingly being investigated. Cell culture derived seasonal influenza vaccines are gaining attention and in November 2012, the first seasonal influenza vaccine manufactured using cell culture technology (Flucelvax, Novartis) was approved by the FDA [7] for adults 18 years of age and older. Very recently, in mid-January 2013, the first trivalent influenza vaccine made using an insect virus (baculovirus) expression system and recombinant DNA technology, Flublok (Protein Science Corporation), was approved for the prevention of seasonal influenza in people 18 through 49 years of age [8]. These recent approvals reflect an important trend in the vaccine industry of adopting modern cell culture manufacturing technologies. This strategy is clearly supported by the public health and regulatory agencies that invested massively over the last five years to promote more adequate responses to emerging infectious disease.

Many other strategies, such as subunit or DNA vaccines [9], have taken advantage of cell culture and modern recombinant DNA developments to overcome the limitations of egg-based production, but one of the most promising approach is the recombinant based virus-like particle (VLP) vaccine [10,11].

Influenza virus-like particles are non-infectious and non-replicating particles, displaying intact and biochemically active antigens required for induction of both humoral and cellular immune responses [12]. Influenza VLPs do not contain genetic material, but rather are empty particles composed of one or both of the two viral immunogenic activators of influenza: Hemagglutinin (HA) and Neuraminidase (NA) [11]. In some cases, influenza VLPs are also constructed with one of the two influenza matrix proteins, either M1 or M2 [10,12,13]. Unlike other DNA based strategies, the presence of NA in influenza VLP constructions is one advantage of this technology, as this antigen was demonstrated to participate in the host protection against influenza infection [14,15].

Different production strategies have been investigated for influenza VLPs. They vary according to the viral strain, the type of gene delivery utilized and the nature of the host-cell expression system. Currently, influenza VLPs are produced in mammalian, insect or plant cell cultures using a variety of vectors and gene delivery techniques [16-18]. Table 1 lists key studies describing the various production methods used to generate influenza VLPs, along with the quantification and purification strategies employed.

**Table 1:** Examples of influenza virus-like particle production

**Influenza viral protein**	**Influenza strain**	**DNA vector system**	**Cell expression platform**	**Purification**	**Characterization quantification**	**Ref**
HA,NA, M1, M2	A/Udorn/72 (H3N2)	rBV	Sf9 insect cells	Partial purification and concentration by ultracentrifugation/Iodixanol and sucrose gradient ultracentrifugation	EM/WB/SDS Page	[19]
HA (H2/H7/H5) NA, M1	A/Indonesia/05/2005(H5N1)	rBV	Sf9 insect cells	Sucrose step gradient ultracentrifugation	EM/Quantitative-WB/SDS Page/SRID assay/Hemagglutination assay	[20]
	A/Swine/Missouri/4296424/2006 (H2N3)					
	A/New York/107/2003 (H7N2)					
	A/Viet Nam/1203/2004 (H5N1)					
HA, NA, M1	A/HongKong/1073/99 (H9N2)	rBV	Sf9 insect cells	Partial purification and concentration by ultracentrifugation/Sucrose gradient ultracentrifugation	EM/Quantitative WB/SDS Page/Neuraminidase activity/Hemagglutination assay	[13]
HA, M1	A/Puerto Rico/8/1934 (H1N1)	rBV	Sf9 insect cells	Partial purification and concentration by ultracentrifugation/Sucrose gradient ultracentrifugation	EM/WB/SDS Page/Hemagglutination assay	[21]
	A/WSN/33					
HA, NA, M1	A/Fujian/411/2002 (H3N2)	rBV	Sf9 insect cells	Sucrose gradient ultracentrifugation/Ion-exchange chromatography	EM/WB/SDS Page/TBA Neuraminidase assay/Hemagglutination assay/SRID assay	[22,23]
	A/Indonesia/5/2005 (H5N1)					
HA, M1	HA: A/California/04/2009 (H1N1)	rBV	Sf9 & BTI-TN5B1-4 insect cells	Partial purification and concentration by ultracentrifugation/Sucrose gradient ultracentrifugation	EM/Quantitative WB/SDS Page/Hemagglutination assay	[16]
	M1: A/Udorn/307/1972 (H3N2)					
NA	A/Cambodia/JP52a/2005 (H5N1)	pCDNA plasmid	HEK 293T – HeLa – A549 mammalian cells	Partial purification and concentration by sucrose cushion ultracentrifugation/Sucrose gradient ultracentrifugation	EM/Quantitative WB/SDS Page/NA Star Neuraminidase assay	[24]
HA, NA, NP, M1, M2, NS2, PB1, PB2, PA	A/Udorn/72 (H3N2)	pCAGGS plasmid	HEK 293T & HeLa mammalian cells	Partial purification and concentration by sucrose cushion ultracentrifugation/Sucrose gradient ultracentrifugation	EM/Quantitative WB/SDS Page/VLP infectivity assay (transfer of GFP-expressing pseudogene)	[25]
HA, NA, M2	A/Chicken/FPV/Rostock/1934 (H7N1)	MLV GagPol	HEK 293T & TE671 human cells	Partial purification and concentration by sucrose cushion ultracentrifugation	Quantitative WB/SDS Page	[26,27]
	A/Thailand/KAN-1/04 (H5N1)					
HA, NA, M1	A/Vietnam/1203/2004 (H5N1)	rMVA vector	Vero monkey cells	Partial Purification and concentration by filtration with 100kDa molecular weight cut off/Sucrose gradient ultracentrifugation	EM/WB/SDS Page/Hemagglutination assay	[28]
	A/Puerto Rico/8/1934 (H1N1)					
HA, NA, M1	A/Puerto Rico/8/1934 (H1N1)	rBV	HEK 293T human cells	Partial purification and concentration by sucrose cushion ultracentrifugation/Sucrose gradient ultracentrifugation/Precipitation by ultracentrifugation	BCA Total protein assay/WB/SDS Page/Hemagglutination assay	[17]
HA, M1	A/Indonesia/5/05 (H5N1)	rAgrobacterium	*N. benthamiana* plant cells	Centrifugation/Affinity chromatography	EM/WB/SDS Page/Total protein analysis Lipid analysis/Hemagglutination assay	[29]
	A/New Caledonia/20/99 (H1N1)					
HA, NA, M1, M2	A/Taiwian/083/2006 (H3N2)	pC14 plasmid	Vero monkey cells	Partial purification and concentration by sucrose cushion ultracentrifugation/Sucrose gradient ultracentrifugation	EM/WB/SDS Page/Total protein analysis/Hemagglutination assay/Dynamic Light Scattering/LC-MS/MS	[12,30]
	A/Hanoi/30408/2005 (H5N1)					

Associated expression system, quantification and purification applied (*rBV, Recombinant baculovirus; MLV, Murine Leukemia Virus*)*.*

Thus far, immunizations with influenza VLPs to protect against either seasonal or pandemic influenza strains have shown promising results [13,31,32]. A general description of the different immunization trials performed thus far is presented in Kang et al. 2009 [33]. Now that proof-of- concept has been established with influenza VLPs as a vaccine candidate, the next step involves the development and scale-up of robust manufacturing processes to provide clinical trials with vaccine doses. Process development and clinical trials are both strongly dependent on the capacity to efficiently characterize and quantify the vaccine candidate using validated methods that are accepted by regulatory agencies. Unfortunately, as detailed in this review, there is still an unmet need for reliable analytical approaches accounting for the intrinsic characteristics of influenza VLPs.

Characterization and quantification methods are needed at different stages during manufacturing process development. Optimization and scale-up generally require rapid, reliable and easy to set-up methods in order to screen a large number of operating conditions. For that reason, the methods in-use should be able to tolerate crude material of each step of a process. Past studies have been essentially focused on the immunogenicity of VLPs as vaccine candidates, with little attention paid to process analytical technology. While most of these investigations have applied standard influenza methods to analyze purified VLP material, quantification methods that can tolerate in-process samples are becoming a necessity to support emerging studies focused on the development of VLP production processes.

Presently there are no total particle quantification methods for influenza VLPs other than estimation by a correlation between the red blood cell hemagglutination assay (HA assay) and total number of influenza particles [34]. Antigen based influenza virus quantification methods (i.e. HA assay, NA assay, SRID) to date have been used on VLPs to characterize purified end product, not to assess process conditions and aid optimization. The applicability of these methods (background, level of detection, level of quantification) in upstream and downstream unpurified samples has not been explored for influenza VLPs, but studies from influenza virus production in MDCK cells have been completed with in-process samples for both the HA and NA assays [1,35].

Therefore, the goal of this review is to present the different challenges researchers face regarding appropriate quantification techniques for influenza VLP vaccines. Specifically, the need for a robust total particle quantification technique that can analyze upstream and downstream samples, the applicability of current influenza quantification methods to assist VLP process development and, with regards to available technologies, what could be proposed for future developments. As a summary, Table 2 presents a comparison of current and future methods available for influenza-VLP quantification.

**Table 2:** Potential or in-use quantification methods for influenza virus-like particles

	**Quantification method**	**Used with flu-VLPs**	**Sample type**	**LOD**	**LOQ**	**Working Range**	**SD (%RSD)**	**Assay Duration**	**Ref**
**HA antigen**	**SRID**	Yes	P, NP,	~3 μg/ml	~3 μg/ml	3.12 – 100 ug/ml	(10%)	~30 h	[36]
	**HA assay**	Yes	P, NP	2 HA unit/50ul	2 HA unit/50ul	2 - 2048 HA units/50 μl	~0,066 %	~5 h	[37,38]
	**LC-MS**	No	P	25 fmol	25 fmol	25 - 400fmol/10ul	*(H1):* 1.14 (3.5%)	n.s.	[39,40]
							*(H3):* 1.24 (5.6%)		
							*(B):* 5.04 (9.7%)		
	**RP-HPLC**	Yes	V	*(H1):* 0.25 ug/ml	*(H1):* 0.75ug/ml	2.5-100ug/ml	3.58 %	10-20 min	[41]
				*(B)*: 1 ug/ml	*(B)*: 3ug/ml		7.73%		
	**Universal ELISA**	No	V		1ug/ml	0.2-16ug/ml	0.14-0.3 (14 - 30%)	n.s.	[42]
	**SPR immuno assays**	No	P, NP, V	<0.5ug/ml	1ug/ml	1-15ug/ml	n.s.	n.s.	[43]
**NA antigen**	**TBA NA assay**	Yes	P	0.34 mU/ml	1.03 mU/ml	0.54 -6.47 mU/ml	2.95%	n.s.	[35]
	**NA star assay**	Yes	P, NP	3 pM	3pM	3-10.5nM	n.s.	~1.5 h	[44]
	**Amplex Red NA assay**	Yes	P	2.09 mU/ml	6.32mU/ml	2.00-7.00 mU/ml	16%		[35]
	**NA-Fluor Assay**	Yes	P, NP	0.09 mU/ml	0.17-2.00 mU/ml	n.s.	2.55%	~4 h	[35]
**HA & NA antigen**	**LC-MS**	No	V	n.s.	n.s.	NA: 2-12ug/ml	n.s.	~5 h	[39]
						HA: 10-70ug/ml			
	**Universal SlotBlot**	No	V, rHA, rNA	0.032 ug/ml	0.032 ug/ml	HA: 0.032-3ug/ml	2300 - 13500 (5%) particles/count	n.s.	[14,45]
						NA: 0.037-10 ug/ml			
**Total particles**	**DLS**	No	P, NP	8.6 × 10^1^ TCI50units/ml	8.6 × 10^1^ TCI50units/ml	8.6 × 10^1^ to 3.4 × 10^4^ TCID50units/ml	n.s.	<30min	[46]
	**Virus Counter**	No	P, NP	*(NP):* 3.6 × 10^6^ VP/ml	*(NP):* 3.6 × 10^6^ VP/ml	10^5^ - 10^9^ VP/ml	0.09 +/− 0.03 VP/ml	~40 min	[47]
				*(P)*: 1.0 × 10^5^ VP/ml	*(P)*: 1.0 × 10^5^ VP/ml				
	**AFFFF-MALS**	No	P, NP	n.s.	n.s.	5 × 10^8^ - 4.5 × 10^9^ VP/ml	n.s.	~1 h	[48-50]

Type of samples: *P*, Purified; *NP*, Non-purified; *V*, Vaccine doses; N.s, Non specified.

### Current influenza VLP quantification methods

Quantification methods for influenza vaccine formulations can be categorized into two general classes. The first provides information on antigen quantity, usually in the form of amount of HA in the vaccine dose (SRID, HA assay) or of enzymatic NA activity, but no commercial vaccine exists with controlled NA. Even though these methods have the ability to provide information on the quantity, functional properties and enzymatic activity of antigens in vaccine doses, they do not directly provide information on how these antigens will elicit an immune response. This is done after vaccination and virus challenge with collected sera coupled with HA inhibition and/or NA inhibition tests. Acknowledging that this is a specific problem that applies to both influenza virus and influenza VLP quantification of final vaccine formulations it will not be further discussed in the following subsections. The other quantification type gives information on whole viral particles, specifically, the total number of particles (qPCR) or the number of infectious particles (Plaque Assay, TCID50).

Quantification of influenza VLPs can also be divided into these two categories; antigen based and whole particle based methods. Antigen based methods are routinely used for the quantification of purified VLP end products, however total particle quantification by qPCR cannot be used for VLP quantification due to their lack of genome. As previously mentioned, there is a relationship between the HA assay and the number of influenza particles that could be used to estimate VLP total particle titers, but this correlation has some caveats that will be discussed. In the following sections, methods that are currently used to quantify and characterize influenza VLPs will be described and discussed in more detail when applied in the context of process development.

### Single radial immunodiffusion (SRID)

Current human influenza vaccine doses are determined from the only validated potency test, the single radial immunodiffusion (SRID) assay. SRID remains the only method to date that has been approved by the WHO authorities for the quantification of HA protein in trivalent influenza vaccines [51]. This method provides an estimation of the antigenicity of the preparation as it is based on standard antigens. It works by measuring the radial diffusion of the viral antigens in an agarose gel containing specific antibodies. In contrast to the hemagglutination assay (described next), this method measures the HA content expressed in μg of HA/ml. It generally has a limit of detection of approximately 3–5 μg/ml [36]. Medicago Inc. is currently undergoing pre-clinical and clinical trials with their plant based influenza VLP candidate against A/California/2009, which has been quantified using the SRID method [52].

Over the years, several limitations of this test have been described in the literature. Firstly, non-aqueous vaccine components can interfere with the diffusion of the HA antigen in the agarose gel [53]. This can pose a significant challenge for quantifying VLPs in unpurified samples taken at different stages during production and purifications steps. Secondly, this method requires a long processing time, has a low sensitivity and needs updating to the homologous HA antigen references and their specific monoclonal antibodies every year [43,54,55]. In the case of a pandemic, this annual update might result in prolonged delays that would prevent rapid vaccine availability. Thirdly, HA protein is prone to aggregation. This effect is particularly relevant for concentrated preparations because high concentrations of influenza viral particles were shown to be more susceptible to aggregate formation [56]. Large aggregates may prevent proper diffusion and interfere with readings. To address these challenges, WHO is encouraging research groups and pharmaceutical industries to develop alternative methods that can provide rapid and reproducible results and replace SRID-based quantification [6]. This is targeted to influenza virus-derived vaccines but could be transposed to VLP vaccines as well. In the case of VLPs however, this method does not resolve the pressing issue of needing a quantification method that can handle in-process material.

### Hemagglutination assay

The hemagglutination assay (HA assay) was the first method proposed to quantify the influenza virus based on its agglutination property [37]. After some modifications from Salk, the method has remained largely unchanged and has been applied for the determination of HA activity in viral preparations [57,58]. The activity measurement is based on the observation of the agglutination of red blood cells (RBCs) by the HA protein. Donald and Issacs established by quantification of viral particles with electron microscopy and based on the red blood cell (RBC) concentration that there is approximately one influenza virus for each red blood cell at the end point of agglutination [34]. Although this assay is not considered a standard method by health authorities, many production and purification yields of influenza-VLPs were evaluated using this technique in the literature (see Table 1).

The principle of this assay is simple, but its preparation is laborious and presents some drawbacks. The red blood cells have to be fresh to obtain reproducible results and their agglutination properties decline over time. Each supply of erythrocyte is different in origin, so assay standardization with an external standard is necessary at each trial. This constitutes a major problem for VLPs, due to the current lack of such standard. No standard protocol is followed for this assay either, as different erythrocyte cell types have been used such as turkey [17], chicken [59] or human [16] at concentrations ranging from 0.5-1.25%. Additionally for wild-type viruses, the host-origin and the influenza strain affect the hemagglutination reaction with the RBCs. Furthermore, this can affect the ratio between total particles and red blood cells as shown by Issacs and Donald [60]. When the virus/RBC = 1 theory was expanded to other viruses containing hemagglutination properties; a discrepancy in the ratio with Influenza C, filamentous influenza virus and the mumps virus was observed. The explanation for this lies in the agglutination ability of these specific viruses. Viruses with increased agglutination ability i.e., increased amount of HA on the particle surface, have a virus to RBC ratio lower than one. Conversely, those with reduced agglutination ability have a ratio higher than one. It has been largely assumed that influenza-VLPs will be morphologically similar to influenza viruses produced in cell culture (spherical in shape, 80–120 nm in size) [12,29,59,61,62]. From electron microscopy images of influenza VLPs produced in our lab, we observed different populations sizes from 100nm to 400nm (data not shown). These VLPs could exert enhanced or reduced agglutination abilities, changing the ratio of VLP to RBC and leading to an under or overestimation for total influenza VLPs. Therefore, to estimate production yield compared to influenza in terms of units of total particles, this method could be useful, but to accurately quantify, more studies need to be completed on the specific relationship between VLPs and RBCs at the agglutination end point.

Another constraint of this assay for VLP quantification is related to the required concentration and purity of the samples, which can limit its applicability for high throughput use during process development. Non-purified samples could give false positives from free protein and contaminating particles from the production system used, as illustrated in Table 3. Table 3 presents the HA titers in mammalian vs. insect cell produced VLPs. For VLPs produced in insect cells, HA was detected in culture supernatants at a level 25 times higher than mammalian VLPs and 4 times less than influenza virus A/PR/8/1934. The main difference between VLP production in mammalian vs. insect cells is the presence of residual baculovirus in the insect cell system [63]. Baculovirus is an enveloped virus that simultaneously buds during VLP production in insect cells and take up HA protein in its viral envelope [64]. The HA assay is unable to differentiate between HA activity from influenza VLPs and HA activity from baculovirus displayed with HA. Thus giving enhanced HA activity readings and total particle estimation. This also poses a problem for other types of VLP quantification. On the contrary, mammalian produced VLPs avoid the problem of baculovirus contamination by using either transient transfection or the bacmam system for VLP production.

**Table 3:** Comparison of HA assay response from VLPs produced in Mammalian and Insect cells and chicken egg derived influenza virus

**Production System**	**Influenza strain**	**Sample preparation**	**HA titer**	**Contamination**	**Normalized Concentration (HA units/ml)**
VLPs from Sf9 - baculovirus infection [16]	HA:A/California/07/2009 (H1N1)	Cell culture media	16 HA units/50 μl	baculovirus 10^8^ BV/ml	16 HA units/50 μl
	M1:A/Udorn/307/1972 (H3N2)				
VLPs from HighFive - baculovirus infection [16]	HA:A/California/07/2009 (H1N1)	Cell culture media	16 HA units/50 μl	baculovirus 10^6^ BV/ml	16 HA units/50 μl
	M1:A/Udorn/307/1972 (H3N2)				
VLPs from HEK 293 T - plasmid transfection [17]	HA/NA/M1: A/Puerto Rico/8/34 (H1N1)	Purified VLPs concentrated 200 times	32 HA units/50 μl	none	0.16 HA units/50 μl
VLPs from HEK 293 T - baculovirus transduction [17]	HA/NA/M1	Purified VLPs concentrated 200 times	128 HA units/50 μl	none	0.64 HA units/50 μl
	A/Puerto Rico/8/34 (H1N1)				
Influenza virus Chicken Eggs [59]	H1N1 A/PR/8/1934	Purified virus	256 HA units/50 μl	none	256 HA units/50 μl

This is a rough comparison, as each system used a different source of red blood cells (chicken, turkey, human) at different percentages (0.5-1.25%).

Nevertheless, for this case, the assay sensitivity is questioned for quantifying supernatant. Mammalian VLP productions had to be concentrated 200 times to reach measurable titers of 32 HA units/50 ul, considering the limit of detection for this technique is 2 HA units/50 ul (Table 2). Additionally, false negatives could arise if sample concentration is not taken into consideration when analyzing supernatants from VLP productions in different production systems. All this information illustrates the main constraint with using the HA assay to aid process development, susceptibility to false positives and insufficient limit of detection.

### Enzymatic NA activity assay

Another approach for VLP quantification specifically applies to those VLPs that include the viral glycoprotein neuraminidase (NA). During a regular infection cycle NA acts as an enzyme that is specifically responsible for the release of viral particles from the cell. Several NA activity assays are commercially available and have been used to evaluate VLPs. VLPs have been tested previously for NA activity using the classic colometric thiobarbituric acid (TBA) method [13,59], a chemiluminescence-based assay using the 1,2-dioxetane derivative of sialic acid as a substrate (NA- star Influenza Inhibitor Resistance Kit, Invitrogen) [24], and two different fluorometric methods (Amplex Red Neuraminidase Assay Kit, Invitrogen) [59] and FL-MU-NANA [65]. Nayak et al. [35] compared different NA assays; TBA, Amplex red and FL-MU-NANA for their ability to quantify in- process crude samples during influenza production in MDCK cells. They found the FL-MU-NANA method to be the most robust with low background in crude samples and high sensitivity, allowing for NA detection as low as 0.09 mU/ml and quantification as low as 0.26 mU/ml, with a range of linearity of 0.17 - 2.00 mU/ml. As an aside, supernatant samples were diluted 4–100 times before assaying. On the other hand, the NA-star system reports 67-fold higher sensitivity [44] compared to the FL-MUNANA system, albeit the NA activity experiments were completed with purified material and the authors report that this method may not be suitable with cell culture media due to interference from the quenching effects of phenol red. Wen et al’s study of VLPs produced in insect cells has shed some light regarding the level of NA activity in VLPs vs. influenza virus; A/PR/8/1934 had 4 times the amount of NA activity of VLPs, which agrees with the levels from the HA assay of Sf9 VLPs vs. influenza virus [59]. Considering that in Nayak et al’s study the virus samples were diluted 4-100 times [35], the FL-MUNANA method has the potential for monitoring NA activity levels in crude and in-process VLP productions. However to date, no studies have been done on this application.

### Total protein analysis

Total protein analysis (protein assays, densitometry by silver stain/coomassie blue and western blot) detect all the proteins present in a sample, which may also include proteins from the host-cell, medium or other viral vector proteins such as those from baculovirus. Due to the lack of specificity, these techniques can only be used for samples containing purified material and do not qualify for use during process development with crude samples. However, they could be good qualitative methods to evaluate the final production yield and purity. Analysis of purified VLPs was usually performed with SDS-PAGE/Western Blot (WB) to determine the presence of specific influenza proteins [22,59]. The relative percentage of antigens in purified VLP samples from Sf9 insect cells showed a composition of 15% HA by coomassie blue staining and 12% by western blot. NA and M1 proteins were seen by coomassie blue, but not measured by densitometry [59]. Mammalian H5N1 VLPs reported 22% and 10.9 % of HA and NA, respectively, for the relative abundance of total VLP proteins [30] by coomassie blue staining measured by densitometry. In another study by Wu et al., using LC-MS/MS they identified 22 VLP associated host cell proteins commonly found in the interior or exterior of influenza virus particles [12]. The LC-MS/MS method and its applicability for VLP quantification will be discussed further in the following section. Protein assays are frequently described in the literature for the quantification of purified VLPs as illustrated in Table 1. D’Aoust et al. demonstrated 80% purity of plant derived VLP produced purified by size exclusion chromatography [29]. Additionally, protein assays such as the bicinchoninic acid (BCA) assay [17] or the Quant-IT assay kit with bovine serum albumin (BSA) [12] were used to quantify the total protein content in purified samples.

### Electron microscopy (TEM)

Historically, electron microscopy (EM) has been widely used for virus observation and has already been applied to influenza whole virus quantification [34,60]. Up to this point, EM has been used to verify the presence and characterize influenza VLPs in terms of morphology and size of the particles [13,16,28].

The main constraints of this technique are sample preparation complexity, the price of equipment and level of expertise needed to analyze and run samples. Moreover, as microscopy counts are based on visual enumeration, samples should be pure enough in order to distinguish the particles, thereby also making this method unsuitable for the analysis of crude samples collected upstream and downstream. Besides contamination from cellular debris, some production strategies have to face contamination with other types of particles, either other viruses used as a gene delivery vectors (i.e baculovirus), aggregates of influenza proteins or vesicles produced by the host cell that may be present in purified material [63]. Currently, there is no other option to validate the physical presence of VLPs in a highly purified sample.

### Potential novel methods for influenza VLP quantification

This section critically reviews the most promising novel analytical approaches with regards to the potential of these techniques to analyze in-process samples for both antigen and physical particle counts. These techniques were used for quantification of either influenza whole-virus or other VLP associated viruses. Technologies based on physical properties instead of biological activity of either antigen or particles are good options in order to have generic, rapid and reproducible VLP quantification. Additionally, they usually have lower limits of detection and broader range of analysis than biological methods, with better selectivity and sensitivity. Potential methods to quantify total particles, such as Dynamic Light Scattering (DLS), Asymmetric Field-Flow Fractionation Using Multi- Angle Light Scattering Detection (AFFFF-MALS); Electrospray Differential Mobility (ES-DMA) and a flow cytometry method (using the Virus Counter) will be discussed for their application to VLPs. For methods dedicated to quantify HA or NA antigens, liquid-chromatography based methods (Reverse Phase High Pressure Liquid Chromatography (RP-HPLC), Liquid Chromatography with Tandem Mass Spectrometry (LC-MS/MS)) are a valuable option, but biological activity is still an important aspect to assess. Immunoassays such as ELISA, Slot Blot, and Surface Plasmon Resonance (SPR) evaluate biological activity and will be further discussed in this section. These methods have the potential to analyze samples from a variety of different matrices and concentrations, such as those during process development. However, more development and validation for their successful application to influenza VLP quantification is still needed.

### Methods based on antigen detection

#### Liquid chromatography methods

**Reversed-phase high performance liquid chromatography (RP-HPLC)** RP-HPLC approach to quantify the hemagglutinin content in influenza vaccines is a very promising technique. It has been investigated since 2006 by two research groups; the Center of Vaccine Evaluation, Health Canada and Solvay Biologicals [41,53,66,67]. Both methods developed quantify the HA1 subunit of HA. Different sample treatments to release HA1 were evaluated, but both groups found the best sample treatment was a two-step process. It includes treatment with detergent (Zwittergent 3–14) to solubilize membrane proteins followed by treatment with a reducing agent (dithiothreitol, DTT) to break the disulfide bounds between HA1 and HA2 subunits [53]. The method was proven to be efficient for quantification of all the 16 HA subtypes from A and B strains [53], for both seasonal and pandemic virus [67]. It is able to handle both egg and cell culture-derived samples [53], but presently its use is limited to the final vaccine formulation or purified material. However, different types of final formulation were tested, and the inactivation agent seems to have an impact on the chromatograms obtained. β-propiolactone creates peak interferences, which changed the retention time (RT) and the peak width of the HA1 subunit whereas formaldehyde-inactivated samples did not show any peak deviation [53].

According to the authors, the method showed high sensitivity. Depending on the strain analyzed, the limit of detection (LOD) could be as low as 0.25 μg/ml of HA which is about 12 times lower than the LOD of SRID assay. The main advantage of this method holds in its fast analysis time and its potential to be used at all process stages, pre- and post-purification.

This method also presents a significant advantage over other techniques, as it can distinguish, in trivalent vaccine formulations, HA content from each HA subtypes in a single analysis (i.e the amount of HA in H1N1 vs. H3N1) based on the retention time [41,53,66]. Kapteyn et al. showed that H1, H3 and B subtypes eluted respectively at 4.8 min, 3.7 min, and 3.2 min. Differences in HA1 retention time were recently attributed to primary structure and glycosylation differences [53].

The type of detection method used also seems to be of importance with regards to sensitivity. Kapteyn et al. show a response of 100 times higher when detecting with a fluorescence (FLD) wavelength (λ = 335nm) compared to UV absorption at a wavelength of 215nm. This is of great importance considering the constraint of low production yields observed for influenza-VLP productions. With both detection methods, response intensity was dependent of the influenza strain of origin for HA1. This is most likely related to the variation in amino acid composition of different HA subtypes. It has been shown that the presence of large amounts of aromatic amino acids such as tryptophan and tyrosine as well as the associated sites of O- and N-glycosylation can modify the signals [53,66].

Reverse-Phase HPLC is a valuable method to implement at the stage of the process optimization and scale-up for influenza VLP production; however, it has to be kept in mind that this method is only quantifying the amount, or mass, of HA protein present in the sample. Although it has already been considered by regulatory authorities as an alternative method to pre-qualify pandemic vaccine lots while the SRID antibodies and standards were still being generated (personal communication with Health Canada and WHO representatives), assessing the antigenicity of vaccine products, through SRID or other anti-HA antibodies binding assays, will still be required for release of clinical influenza vaccine lots.

**LC-MS methods** Recently, LC-MS and LC-MS/MS methods have demonstrated the ability to quantify both HA and NA content in influenza vaccine formulations from different origins, egg- or plant-derived [39,40]. Evaluation of the LC-MS/MS method was performed on different sample types, including seasonal trivalent influenza vaccines containing H1N1, H3N2 and B strains, monovalent pandemic vaccines, vaccine bulk preparations and recombinant antigens. The method was described as rapid, providing quantification of all protein components in a single analytical procedure, and above all, did not depend on the availability of specific antibodies or standard antigens [39]. The principle lies on analyzing the mass and charge of peptides generated from protein trypsinization. To quantify the amount of target protein, the combined signal from the three most intense peptides is compared against an internal standard. Limits of detection and of quantification are 1 μg of HA or NA protein, which falls in the lower range of the quantification methods previously discussed. Williams et al. [40] argue that this level could be as low as 150pg of protein when considering classic levels of detection for LC-MS/MS.

Similarly to HPLC-based methods, this method does not measure the immunogenic activity of the HA antigen such as the SRID method. Therefore, it can only be used as a method to quantify antigen content in vaccine formulations. One of the interesting points of this study is the discrepancies highlighted when compared to vaccine doses measured by SRID for different strains in the seasonal vaccine formulations. The SRID method reported HA levels three times higher than HA protein content quantified by the LC-MSE (LC-MS with an elevated energy acquisition method) physical direct method. These results clearly demonstrate the failure of SRID standardization method.

The authors cite critical factors on which the method’s success depends. One critical factor is to insure that trypsin digestion is performed at its maximum capacity and that sufficient quantity of protein is available to release a detectable amount of tryptic peptides. This is a very important roadblock with regards to VLPs quantification considering the low production levels achieved thus far.

Another issue is the composition of the sample matrix. Obviously, purified material is the ideal material to work with, avoiding interference with contaminant proteins from the cell culture supernatant. When LC-MS/MS was used for influenza virus vaccine doses produced from eggs it was able to identify and quantify up to 19 μg contaminant protein/vaccine dose. A method that is able to quantify both protein of interest and contaminants at the same time has a clear advantage over other methods from a regulatory standpoint. Nevertheless, it also shows that sample matrix will have a strong effect on the number of peptides identified.

### Immunoassays

The identification of universal antibodies against HA and NA proteins has been an important breakthrough for quantification techniques based on biological activity. A recent development for the Slot Blot and ELISA assays, which aims to develop a method that can analyze all current and new circulating strains of influenza, is very promising for the quantification of influenza proteins and VLPs. Rabbit polyclonal antibodies have been raised against highly conserved regions of all the subtypes of influenza A and B hemagglutinins and neuraminidases [14,42]. In the case of HA, universal antibodies were raised against a sequence of 11 amino acids in the N-terminal region of HA2. This amino acid sequence is the most broadly conserved region among all influenza A subtypes [42]. For NA, the regions selected were located either (i) close to the enzymatic site of NA (ILRTQESEC), or (ii) in the cytoplasmic tail at the N-terminal of the neuraminidase (MNPNQKIITIGS) [14]. Universal antibodies raised against HA2 were found to detect one B strain and the 13 different subtypes of A influenza strains while universal antibodies raised against the NA conserved regions reacted against the 9 subtypes of NA, in both avian and human influenza strains.

The use of these antibodies for in-process samples has not been validated yet for production with cell culture based systems but no cross-reactivity with egg proteins was observed for uninfected or infected allantoic fluids [14,42]. Additionally, universal antibodies are also able to detect recombinant proteins produced in insect-cells.

**Enzyme-linked immune sorbent assay (ELISA)** Based on the anti-HA universal polyclonal antibody, a competitive ELISA assay was recently published [42]. Authors demonstrate that the new quantification method of both recombinant HA and human vaccine provided very similar values to those obtained by the SRID assay. This method is very promising for future vaccine candidate development especially in the case of future pandemic vaccines. However, it still needs further development to define LOD and LOQ to be able to compare with traditional SRID assay.

**Slot-blot** Another quantification technique based on universal recognition of HA or NA antigens, is the Slot blot technique [45,68]. The authors argue in favor of this technique by presenting it as a simple, highly reliable, inexpensive and easy to operate technique. The procedure is fast, only taking 5 h. However, this assay was far less documented than the proposed techniques previously cited. No indication of the precision or repeatability of the method was provided. Consequently, at that stage, it is still not possible to compare its efficiency compared to classic neuraminidase enzymatic activity techniques or the SRID assay.

**Surface Plasmon Resonance Immunoassay (SPR)** The surface plasmon resonance (SPR) was also proposed as an alternative to the SRID assay [43]. This technology has been originally commercialized under the name of Biacore® [69]. The principle is based on the competitive binding of specific anti-HA antibodies between HA protein present in the sample and rHA protein coated on the gold biosensor surface. The signal measured is inversely proportional to the concentration of HA protein content in the sample. An influenza quantification with SPR technology using low affinity ligands, e.g., lectins or specific carbohydrate structures, such as sialic acids that bind HA protein on the biosensor surface was tested [70]. This technique was abandoned due to low limit of detection and then was further developed using antibodies. However, it might be advantageous to pursue the development of this approach using a ligand binding system that “recognizes” different influenza virus strains eventually leading to a more universal method.

This technology offers higher sensitivity in different types of sample matrices and precision than the SRID method, with a limit of detection as low as 0.5 μg/ml for A/H3N2 Wisconsin/67/2005, A/H1N1/Solomon Islands/3/2006 and B/Malaysia/2506/2004 strains [43] and limit of quantification of 1 μg/ml. The specificity of the sera from sheep origin for the recombinant protein used was tested, and the authors found that the responses were specific to their matching recombinant protein type with no cross reactivity. However, serum from chicken A/H1N1/PR/1934 was non- specific and bound to each recombinant protein matrix. Samples were analyzed with the Biacore® at different stages of the vaccine purification process, from cell culture supernatant harvest to ultra/dia-filtrated samples [43]. It was found that the Tween 20 surfactant contributed to prevent adsorption of proteins to the matrix and HA aggregation. Increasing sucrose (>1%) and NaCl (>0.2M) concentrations resulted in a decreased response, thereby increasing the calculated amount of HA present in the sample. For in-process VLP samples this shouldn’t pose a problem, but for partially or fully purified samples by either sucrose cushion and gradient ultracentrifugation or chromatography that routinely have higher levels of sucrose and NaCl greater than 20% and 0.5M, respectively, lower responses might be observed.

### Methods based on total particle quantification

**Virus counter (cytometry)** A focused flow cytometry method [71], the Virus Counter has recently been proposed as a method to quantify total virus particles. Previous studies have been completed with influenza virus [47]. The basic analytical principle exploited in the Virus Counter relies on flow cytometry and fluorescence detection. The Virus Counter uses a non-virus specific dye, Combo Dye, which stains proteins (red) and nucleic acids (yellow). It is equipped with software that quantifies intact virus particles, those that emit orange fluorescence, which contain both labeled proteins and nucleic acids. This dye is advantageous compared to traditional antibody labeling techniques that can be laborious and expensive. Additionally, it solves the problem of the requirement for specific strain influenza antibodies that impede vaccine development. This method has a quick and simple sample preparation and runtime, which includes 30 minutes incubation with the dye at room temperature and 10 minutes analysis time. Stepp et al [47] compared quantification by the Virus Counter to qPCR for total particle determination of H1N1 A/California/2009 and also to the manufacturer’s TCID50 and TEM values. They found that all values correlated significantly with each other. Ferris et al. completed a similar study for baculovirus quantification, comparing the Virus Counter to the plaque assay (PFU) method using non-purified material [72]. They found the limit of detection increased approximately 1 log when using non-purified samples. While there have been no studies done with VLPs to date, the Virus Counter seems to be a promising method for VLP quantification during process development considering its low limit of quantification even in non-purified samples. However, because the system has been set up for virus quantification, the dye and software is tailored for labeling and detecting virus samples (protein + nucleic acid). While this system could still provide information on the quantity of VLPs that contain host cell derived nucleic acids, a concern for regulatory agencies, quantification could be inaccurate if it does not include protein-only detection. Therefore, either the software would need to be adapted for VLP quantification or the method used to label VLPs changed, potentially labeling NA or HA proteins with red and yellow fluorescent tagged antibodies. This would bring up the problem of strain specific antibodies, so staying with a generic labeling system with adapted software would be the most beneficial to address the need of total particle quantification for VLP process development and final product characterization.

### Size-based techniques

**Dynamic light scattering (DLS) with gold nanoparticles** Light scattering technologies, measuring the size, the aggregation and the zeta potential of particles has already been evaluated for the detection and quantification of different viruses or VLPs [46,73-75]. Wu et al and Pincus et al. used DLS to analyze the average size of purified influenza VLPs, but not to quantify total particles [12,51]. One recent paper has applied this technique to influenza detection [46]. The principle is based on influenza specific antibodies conjugated with gold nanoparticles (AuNP) used as probes. The aggregation between these probes and the virus is measured by DLS technology, and the mean hydrodynamic diameter (DH) of the formed aggregates is correlated to virus concentration. AuNP light scattering is highly efficient compared to light scattering from biomolecules [76,77] therefore when coupled with specific binding to VLPs this method presents the possibility of high sensitivity with low background, even with crude samples. However, a level of background from contaminating particles from the production system such as vesicles or baculovirus would have to be established. The detection limit is 1–2 folds lower than the traditional infectious particle count technique, TCID50 assay, with a value of 8.6 × 10^1^ TCI50 units/ml, with an increase in DH with increasing concentration of influenza up to 3.0 × 10^4^ TCID50 units/ml. DH decreases at concentrations higher than 3.0 × 10^4^ TCID50 units/ml from a phenomena known as the hook effect [78]. Above this concentration, the virions are in excess of AuNP, resulting in a decreased mean DH. One important consideration for this method is that the antibody chosen for conjugation to AuNP must be able to bind to whole intact virus, or VLP, and not regions that are only exposed after particle disruption or protein cleavage (i.e. the conserved stalk region of HA2). Another is the presence of contaminating particles from the production system such as vesicles that could alter the mean DH. AuNP probe concentration and size also have an impact on the technique detection limit and would need to be optimized for influenza VLP application. A comparison of detection of influenza viruses produced in eggs or MDCK cells proved that the technique is not sensitive to change in the sample matrix. Therefore, DLS is a promising technique to fulfill the lack of total particle count in the case of influenza VLPs and could have the potential to be used to process development with upstream and downstream samples. However; again it still needs be optimized further as this paper does not present any values of quantification errors and a calibration curve with quantified VLPs would need to be established.

**Asymmetric flow field-flow fractionation using multi-angle light scattering detection (AFFFF-MALS)** AFFFF is another size-based technique to separate particles. In this case, the range considered is 1 nm to 100 um. The standard detector used is UV at 280nm, but if it is coupled with a multi-angle light scattering (MALS) detector selectivity could be further increased [48]. The principle is complex but is well described by Pease et al [49]. Briefly, hydrated virus particles are injected into a separation channel containing a porous membrane along the bottom of its surface. Fluid enters the channel, spreading the particles along its width, while liquid is evacuated through the porous membrane. Particle elution is then performed with laminar flow across the channel while a cross-flow is maintained through the membrane. This allows for small particles to elute into the laminar flow channel before large particles. In order to elute remaining large particles and aggregates, cross-flow is stopped thus releasing them into the laminar flow channel.

AFFFF-MALS was successfully applied to H1N1, H3N2 and B influenza strains [50] with both crude and purified material from egg-based production. The range of linearity obtained for B/Yamanashi/166/98 influenza viral strain was of 5 × 10^8^ to 4.5 × 10^9^ VP/ml. One of the main problems occurring when choosing a size detection method for quantifying influenza virus is particle aggregation. By optimizing the equation model, Mc Evoy et al. [48] managed to reach values in very good agreement with TEM for the quantification of a B/Yamanashi/166/98 influenza viral strain (2.2 × 10^10^ VP/ml & 1.6 × 10^10^ VP/ml, respectively). The technique also compared the EM and qPCR for total particle counts, and TCID50 or fluorescent focus assay (FFA) [49,50] for infectious counts. Considering the similarity between values obtained by AFFF-MALS and the EM-based method, it is possible to envisage this technique as a reliable, and cost-effective tool to quantify content of total particles in virus and virus-like particle preparations. However, the equipment needed and high level of technicality required still impedes the routine use of this method.

**Electrospray differential mobility analysis (ES-DMA)** The principle of virus quantification from differential mobility analysis (DMA) is analogous to mass spectrometry, which separates peptides on a charge-to-mass ratio, while with DMA particles are separated and analyzed by an ion mobility analyzer on the basis of charge-to-size ratio [79]. Particle separation is completed in the gas-phase after aerosolization of the samples. The particle detection is done either by transmission electron microscopy or with a particle counter after real-time condensation of separated fractions of the samples. The technique is able to separate particles of sizes ranging from sub-nanometer (proteins), to intact viral particles (25-300 nm). To date 21 different types of virus have been assessed with this method. Most of them were non-enveloped viruses, but three enveloped viruses have been successfully analyzed, the Sendai rodent virus, alpha virus, and the murine hepatitis virus. This method has never been applied to influenza virus itself, but it was recently applied to polyomavirus VLPs carrying 17 residues of HA protein from avian influenza strain [49,79].

The main advantage of the ES-DMA technique is the wide linearity range, reaching five orders of magnitude in some cases, with low limits of detection and of quantification (10^8^ VP/ml for phage PP7). It is also able to provide a size distribution of the viral particles, detecting some changes of 0.3 nm [80]. In the case of VLPs, beyond just quantifying the number of particles, characterization of the particles could be of great interest for the field. Consequently, even though it has mostly been developed for non-enveloped small size viruses (20 nm) this technique is a potential interesting tool for influenza-VLP quantification and characterization. Nevertheless, at this stage of development of the method, there are still some drawbacks such as the complexity of operation, and the expertise and equipment required. Another drawback for the application of this technology to influenza-VLP is the challenging step of electrospraying enveloped virus. Optimizing the best operation conditions for this step is still a matter of trial and error as quantitative and theoretical models to describe the impact of electrohydrodynamic forces on lipids envelopes are not yet available [80].

## Conclusion

Until now VLP studies have been completed with the final goal of evaluating their potential as vaccine candidates. Therefore, the current methods available for influenza vaccine quantitation have been sufficient for concentrated and purified final material. However, as more research is done on influenza VLPs through the lens of bioprocessing for industrial applications; it has become obvious that these methods are deeply impeding the development of manufacturing processes. Not only do they come with problems already associated with influenza quantification (lack of universal standard and a direct total quantification method), they are also laborious and cannot be applied for analysis of in-process samples. Potential quantification methods for both antigen and total particles were presented in this review that fulfills most of these needs (low limit of detection, in- process use). For some of them the main drawback remains their complexity of operation, which is not necessarily compatible with an application for process monitoring or process development phases. Nevertheless, we are on the way to develop usable techniques that are compatible with different production platforms and applicable for process development and optimization studies for influenza VLPs.

## Competing interests

The authors declare that they have no competing interests.

## Authors’ contributions

CMT and EP, contributed equally to the literature research, analysis and interpretation of the literature data as well as writing of the final review. AL contributed to the design of the study, analysis of data and drafting of the original manuscript. OH contributed to drafting and revising the manuscript critically for important intellectual content. AAK made substantial contributions to conception, design, and critical revision of the manuscript and gave final approval of the version. All authors read and approved the final manuscript.
